# Evidence for a Role of *LPGAT1* in Influencing BMI and Percent Body Fat in Native Americans

**DOI:** 10.1002/oby.20243

**Published:** 2012-07-26

**Authors:** Michael T Traurig, Julieanna I Orczewska, Daniel J Ortiz, Li Bian, Alejandra M Marinelarena, Sayuko Kobes, Alka Malhotra, Robert L Hanson, Clint C Mason, William C Knowler, Clifton Bogardus, Leslie J Baier

**Affiliations:** Phoenix Epidemiology and Clinical Research Branch, National Institute of Diabetes and Digestive and Kidney Disease, National Institutes of HealthPhoenix, Arizona, USA

## Abstract

**Objective:**

A genome-wide association study (GWAS) was recently completed in 1120 Pima Indians to identify loci that influence BMI. Among the top 100 signals were three variants that mapped within the *lysophosphatidylglycerol acyltransferase 1* (*LPGAT1*) gene. LPGAT1 belongs to a large family of acyltransferases, which are involved in a variety of biological processes including pathways that regulate energy homeostasis and body weight. Therefore *LPGAT1* was analyzed as a candidate gene for obesity in Pima Indians.

**Design and Methods:**

Variants (*n* = 26) located within and adjacent to *LPGAT1* including a novel 27bp deletion in the 5′-untranslated region identified by sequencing were genotyped in a population-based sample of 3,391 full-heritage Pima Indians living in the Gila River Indian Community. Replication of selected variants was assessed in a second sample of 3,327 mixed-heritage Native Americans from the same community.

**Results:**

Variants with nominal associations with BMI in each of the two independent samples (tagged by rs112662024 and rs12058008) had associations of *P* = 1-4 × 10^−5^ in the combined sample (*n* = 6718). A haplotype that includes the novel 27bp deletion, which does not occur in Caucasians, showed the strongest association with BMI in the full-heritage Pima Indians. *In vitro* functional studies provided suggestive evidence that this 27bp deletion may affect transcriptional or posttranscriptional regulation. Analysis of *LPGAT1* cDNA from human preadipocytes identified an additional exon whose sequence could potentially serve as a mitochondrial targeting peptide.

**Conclusions:**

LPGAT1 is a novel gene that influences BMI in Native Americans.

## Introduction

The Pima Indians of Arizona have a high rate of obesity (1) and we have recently completed a genome-wide association study (GWAS) using the Affymetrix Genome-wide Human SNP Array 6.0 in 1,120 Pima Indian subjects to identify genes that influence BMI (2). Among the top 100 variants associated with BMI, three variants were located within a region encompassing the *lysophosphatidylglycerol acyltransferase 1* (*LPGAT1*) gene (*P* ≈ 10^−4^ ref. 2). LPGAT1 is part of a large group of acyltransferases and belongs to the lyosphosphatidic acid acyltransferase family, which also includes glycerol-3-phosphate acyltransferases 1-3, lysophosphatidylcholine acyltransferases 1-2 (LPCAT1-2), and various lyosphosphatidic acid acyltransferases (3). Acyltransferases catalyze the transfer of an acyl group, and are involved in a broad range of metabolic pathways including fat storage, food intake, insulin sensitivity, energy homeostasis (4), and the biosynthesis of lipids (5). Knockout studies in mice suggest that several acyltransferases (e.g., *GPAT*s, *DGAT1*/*2*, *CPT1*, *MGAT2*, and *AGPAT6*) may play a crucial role in body weight regulation (6-,7,8,9,10,11,12,13,14). Yang et al. (15) showed that LPGAT1 is localized to the endoplasmic reticulum and may be involved in the reacylating (remodeling) of lysophosphatidylglycerol (LPG) back to phosphatidylglycerol. Hiramine et al. (16) demonstrated that LPGAT1 can also display monoacylglycerol acyltransferase activity. MGATs are enzymes involved in the synthesis of diacylglycerol—a precursor of triacylglycerol. They also observed that targeted knockdown of *LPGAT1* in the liver of *db*/*db* mice resulted in a slight decrease in body weight. Based on the associations from our GWAS with BMI and the potential role of *LPGAT1* in lipid metabolism and body weight regulation, *LPGAT1* was analyzed as a candidate gene for obesity in the Pima Indians of Arizona.

## Methods and Procedures

### Subjects and phenotypes

Characteristics for the individuals and specific exams included in this study are provided in Supplementary Table S1 online. The subjects are part of a longitudinal study (1965-2004) of the etiology of type 2 diabetes in the Gila River Indian Community in Central Arizona (17). Subjects were invited to participate in a health exam every 2 years, which included a 75-g oral glucose tolerance test to determine diabetes status along with measures of height and weight to determine BMI. Only individuals who had at least one measure of BMI at ≥15 years of age were included in this study and exams at which the subject was pregnant were excluded. The full-heritage Pima Indian population-based sample consisted of all individuals whose heritage was reported as full Pima and/or Tohono O'odham (a closely related tribe) (*n* = 3,391). The mixed-heritage population-based sample consisted of all of the remaining individuals who had been studied in the Gila River Indian Community (*n* = 3327). Their reported heritage on average was one-half Pima and one-third American Indian, which may include other tribes. Since many of these longitudinally studied subjects had multiple exams in which BMI was measured (Supplementary Table S1 online), we analyzed both the maximum BMI recorded from any single exam and repeated measures of BMI, which used BMI data from all exams. A subset of these subjects (*n* = 555) was additionally studied as inpatients in our Clinical Research Center when they were nondiabetic. These individuals underwent tests to measure quantitative traits related to obesity such as percent body fat. Some of these subjects took part in multiple exams. In this study, we analyzed both percent body fat measured at a single exam (the exam when the subject was the youngest was selected) as well as repeated measures of percent body fat which included measures from all visits (Supplementary Table S1 online). Body composition was estimated by underwater weighing until January 1996, and by dual energy X-ray absorptiometry (DPX-1; Lunar Radiation, Madison, WI) thereafter. A conversion equation derived from comparative analyses was used to make estimates of body composition equivalent between the two methods (18). All of the subjects provided written informed consent before participation in the study. This study was approved by the Institutional Review Board of the National Institute of Diabetes and Digestive and Kidney Diseases (NIDDK).

### Sequencing and genotyping

To identify potential novel variants in *LPGAT1*, all of the coding exons, exon-intron boundaries, 5′- and 3′-untranslated regions (UTRs) and the promoter regions of LPGAT1 /uc001hiu.1 (isoform A) and LPGAT1/uc001hiv.1 (isoform B) were sequenced in 24 unrelated (not related by the first degree) full-heritage Pima Indians. To determine whether the novel 5′-UTR 27bp deletion (LPAGT1-4) is unique to Native Americans, a DNA panel (Human Random Control Panel-5; Sigma-Aldrich, St. Louis, MO) consisting of 96 randomly selected unrelated Caucasian subjects was also sequenced. Sequencing was done using a Big Dye terminator kit (Applied Biosystems, Foster City, CA) on an automated DNA capillary sequencer (model 3730; Applied Biosystems). Genotyping was done by using either the SNPlex genotyping System 48-plex (Applied Biosystems) on an automated DNA capillary sequencer (model 3730; Applied Biosystems), the Illumina BeadXpress System (Illumina, San Diego, CA), or by allelic discrimination polymerase chain reaction (PCR) (Assays-on-Demand SNP Genotyping products; Applied Biosystems). Genotyping data underwent quality control assessment, which required a successful call rate of >85% of all samples, a deviation from Hardy-Weinberg equilibrium of *P* > 0.001, and a discrepancy rate of <2.5% for blind duplicate samples (330 and 100 blind duplicated samples in the full-heritage Pima Indian and mixed-heritage population-based samples, respectively).

### Statistical analyses

Statistical analyses were performed using the statistical analysis system of the SAS Institute (Cary, NC). For continuous variables, linear regression models were used to analyze the association between BMI and genotype with adjustments for covariates including age, sex, and birth year. The generalized estimating equation procedure was used to account for family membership since some of the subjects were siblings. The logarithmic transformation of BMI was used in these analyses to reduce skewness. In the mixed-heritage Native American sample, the individual estimate of European admixture was also used as a covariate. These estimates were derived from 32 markers with large differences in allele frequency between populations (19) using the analytical method described in Hanis *et al.*(20). A combined test of association for the full-heritage Pima Indian and mixed-heritage Native American samples was conducted by the inverse variance method (21). For the repeated BMI and percent body fat measures, the SAS MIXED procedure which fits a linear mixed effects model using a maximum likelihood method was used for the association analyses (described in ref. 2). Six haplotypes could be defined by the five variants shown in Table 1. Haplotype analyses were conducted using a modification of the zero-recombinant haplotyping method (22), as previously described (23). In brief, the MLINK program was used to assign haplotype probabilities for each individual based on their genotypes, the genotypes of their relatives, and the population haplotype frequencies. These probabilities were then used in regression models, in a fashion analogous to the analysis of single variants, to assess the association with each individual haplotype in comparison with all others. Linkage disequilibrium (LD: *D*′ and *r*^2^) was analyzed using the Haploview program (Haploview http://www.broad.mit.edu/mpg/haploview/). Mixed effects models were used to assess an additive model of association between the *LPGAT1* haplotypes and *LPGAT1* gene expression; age, sex, and Pima heritage were included as fixed effects with relatedness treated as a random effect.

**Table 1:** Association results for the five tag variants with BMI in the full-heritage Pima Indian, mixed-heritage Native American, and combined sets

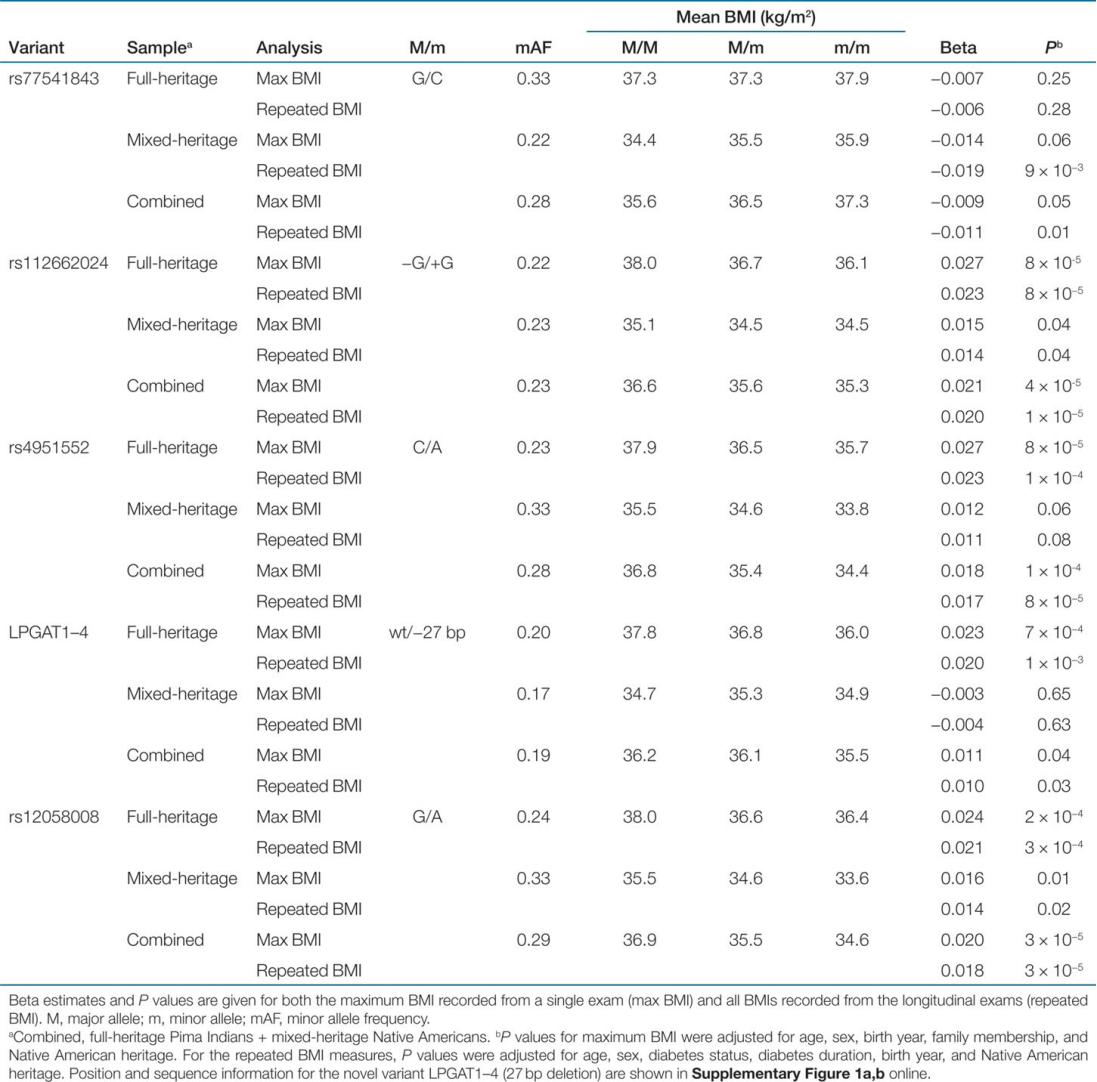

### Plasmid constructs

Genomic fragments with or without the rs112662024 G insertion located in the 3′-UTR of *LPGAT1* were amplified by PCR using DNA from a known heterozygous individual as a template. The fragments (402 bps, chr1:209,989,461-209,989,862, NCBI build 36.1, UCSC Genome Browser) were cloned into the pGL3-promoter vector (Promega, Madison, WI) at the *Xba*I and *Fse*I sites located at the 3′ end of the luciferase gene, which will allow the fragments to be transcribed as the 3′-UTR of the gene. To examine whether the 27bp deletion (LPGAT1-4) located in exon 1 (encodes the 5′-UTR) of LPGAT1/uc001hiv.1 (isoform B) may affect transcription of isoform A or isoform B, genomic fragments with and without the deletion were amplified by PCR using genomic DNA from a known heterozygous individual. The inserts (isoform A: 781 and 754 bps, chr1:210,069,986-210,070,766; isoform B: 600 and 573 bps, chr1:210,070,530-210,071,129, NCBI build 36.1, UCSC Genome Browser) were cloned into the promoterless pGL4.17 vector (Promega) at the multiple cloning region. To examine whether the 27bp deletion may affect gene expression posttranscriptionally, the 5′-UTRs of *LPGAT1* isoform B (RNA accession, NM_014873.1; UCSC ID, uc001hiv.1) and LPGAT1.cApr07 (AceView gene models) with and without the deletion were cloned into the pGL3-promoter vector at the *Hind*III and *Nco*I sites located at the 3′ end of the SV40 promoter and 5′ end of the luciferase gene, respectively. Inserts were amplified by PCR using human cDNA from an individual heterozygous for this variant. All constructs were verified by sequencing.

### Cell transfections and luciferase assays

HepG2, Caco-2, 3T3-L1, and G8 myoblasts were all purchased from American Type Culture Collection (ATCC, Manassas, VA). The HepG2 and Caco-2 cells were maintained in Eagle's Minimum Essential Medium (ATCC) supplemented with 10 and 20% fetal bovine serum, respectively (ATCC). The 3T3-L1 cells were cultured in Dulbecco's Modified Eagle's Medium (ATCC) supplemented with 10% calf bovine serum (ATCC), whereas the G8 mouse myoblasts were cultured in Dulbecco's Modified Eagle's Medium (ATCC) supplemented with 10% fetal bovine serum (ATCC) and 10% horse serum (ATCC). All transfections were done with either Lipofectamine 2000 or Lipofectamine LTX and PLUS Reagents (Invitrogen, Carlsbad, CA) following the manufacturer's instructions using serum and antibiotic free medium. To control for transfection efficiency, co-transfections were done using the pGL4.74[*hRluc*/TK] plasmid (Promega) and normalized to the *Renilla* luciferase expression. Forty-eight hours after transfection the cells were washed with phosphate buffer solution and cell lysates were prepared using the lysis buffer from the Dual-Luciferase Reporter Assay System (Promega). Luciferase activities were measured using the Dual-Luciferase Reporter Assay System. Luciferase activity is expressed as the relative activity and is shown as the means ± s.d. for six replicates.

### Isolation of preadipocyte cDNA and screening for the 27bp 5′-UTR deletion

Preadipocytes were previously obtained from subcutaneous abdominal adipose tissue biopsies as described (24,25) and total RNA was extracted using an RNeasy Mini Kit (Qiagen, Valencia, CA). To remove any residual DNA, the purified RNA was treated with DNAse using RNAse-free DNAse Set (Qiagen). First-strand cDNA was synthesized from preadipocyte total RNA using a BD Advantage RT-for-PCR Kit (BD Bioscience/Clontech, Mountain View, CA) following the manufacturer's instructions. Primers located in exons 1 and 2 of isoform B (RNA accession, NM_014873.1; UCSC ID, LPGAT1/uc001hiv.1) were designed to screen the preadipocyte cDNA for the presence of the 27bp deletion. Primer sequences were as follows: forward, 5′-GCCCTT GGGGACCGAGTCTC-3′ and reverse, 5′-TAGCAACCAGGTTGTTGACGACCA-3′. Since exons 1 and 2 are located in a GC rich region, a GC PCR kit (BD Bioscience/Clontech) was used for the PCR reactions. PCR amplified products were separated by gel electrophoresis using a 2% super fine resolution agarose gel (Amresco, Solon, OH), gel purified using a QIAquick gel extraction kit (Qiagen), and verified by sequencing.

## Results

### Identification of novel variants and association analysis

Sequencing of both *LPGAT1* isoforms A and B in 24 unrelated Pima Indians detected several variants previously identified in other ethnic groups (i.e., variants found in dbSNPs build 135, data not shown) and four novel variants including one in exon 1 (5′-UTR) of isoform A, two in the promoter region of isoform A, and a 27bp deletion (LPGAT1-4) in exon 1 (5′-UTR) of isoform B (locations and sequence information for the four novel variants is provided in Supplementary Figure S1a,b online). Several variants (*n* = 26) including 7 detected by sequencing (the 27bp deletion and 6 previously identified variants), 9 from the initial GWAS, and 10 tag SNPs from the CHB HapMap (minor allele frequency ≥0.1 and a pairwise *r*^2^ ≥ 0.8) spanning a 197-kb region encompassing the *LPGAT1* gene were selected for genotyping in a population-based sample of 3,391 full-heritage Pima Indians. The LD patterns (*D*′ and *r*^2^) for the 26 variants are shown in Supplementary Figure S1 online. Several variants located in a LD block that spans *LPGAT1* and its upstream 5′ region were associated with BMI in the full-heritage Pima sample (Supplementary Table S1 online). Polymorphisms within this block with allele frequencies >1% could be tagged by five variants (Supplementary Figure S1 online). Four of these five tag variants were associated with BMI in the full-heritage Pima Indian sample (Table 1). Defining BMI in these longitudinally studied subjects as either maximum BMI (the maximum BMI on record) or repeated BMI measures (BMI from all longitudinal exams, ranging from 1-20 exams per individual) provided similar levels of associations (Table 1). Among the 555 nondiabetic subjects who had been characterized for traits related to obesity, the 4 variants showing the strongest evidence for association with BMI in the full-heritage Pima sample also had the strongest associations with percent body fat and repeated percent body fat measure (Supplementary Table S1 online). Genotyping of the five tag variants in a second sample of mixed-heritage Native Americans (*n* = 3327) showed that four of the variants had a nominal association with BMI (Table 1). The strongest independent replications were with rs11266202 and rs12058008, and combining the full-heritage and mixed-heritage population sets provided the strongest evidence for association with BMI (Table 1). However, rs77541843 which had no association with BMI in the full-heritage Pima sample had the strongest association in the mixed-heritage sample, and LPGAT1-4 (27bp deletion) was associated with BMI in the full-heritage sample, but not in the mixed-heritage sample (Table 1).

As would be predicted from the single variant association data, the LD (*r*^2^) between the five variants in Table 1 differed in the full-heritage Pima Indians compared with the mixed-heritage Native Americans due to ethnic differences in allele frequencies (Supplementary Figure S1 online). In particular, the 27bp deletion (LPGAT1-4) located in the 5′-UTR has a 20% frequency in full-heritage Pima Indians, but is not annotated in any public database, and sequencing of this region in 96 unrelated Caucasians did not find the 27bp deletion in any of the individuals. These five variants produce six haplotypes (Table 2). Haplotype 2 had the strongest association with BMI in the full-heritage Pima Indian sample, whereas haplotype 1 had the strongest association in the mixed-heritage sample. Haplotypes 1 and 2 differ only at the 27bp deletion (Table 2).

**Table 2:** Haplotype analysis for the *LPGAT1* variants with BMI

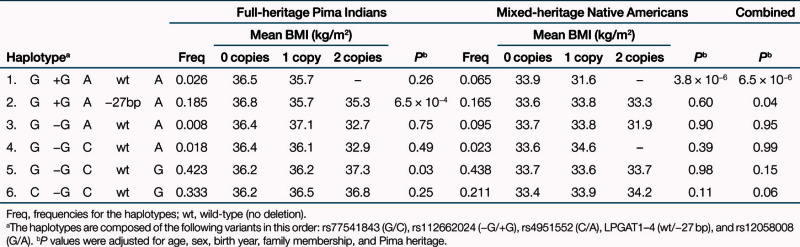

**FIGURE 1:**
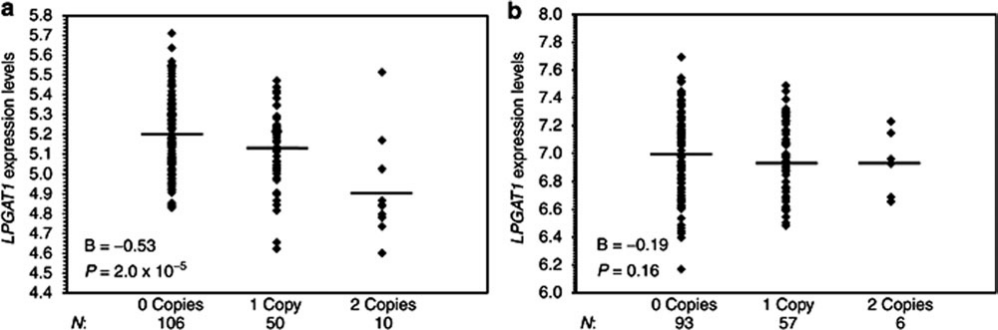
*LPGAT1* expression levels based on the copy number of haplotype 2 in (**a**) muscle and (**b**) adipose. Means are indicated by the horizontal lines. *P* values were adjusted for age, sex, and Pima heritage. LPGAT1, lysophosphatidylglycerol acyltransferase 1.

To determine whether any of the haplotypes could have functional consequences, we looked for an association between each haplotype and *in vivo LPGAT1* gene expression in muscle and adipose tissues. Using genome-wide expression data that had previously been obtained from full-heritage Pima Indians (26), we found that only haplotype 2 was associated with *LPGAT1* expression levels in muscle (Table 3). Individuals with one copy of haplotype 2 had lower *LPGAT1* expression than individuals with no copies of the haplotype, and individuals with two copies of haplotype 2 had the lowest levels of expression (Figure 1a). Although a similar trend of *LPGAT1* gene expression based on copies of haplotype 2 was observed in adipose tissue, the association was not significant (Figure 1b).

**Table 3:** Haplotype analysis for the *LPGAT1* variants with BMI

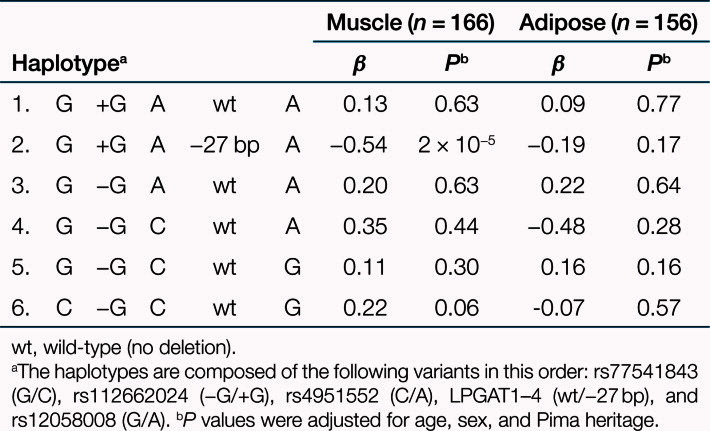

### Identification of an additional exon in *LPGAT1*

**FIGURE 2:**
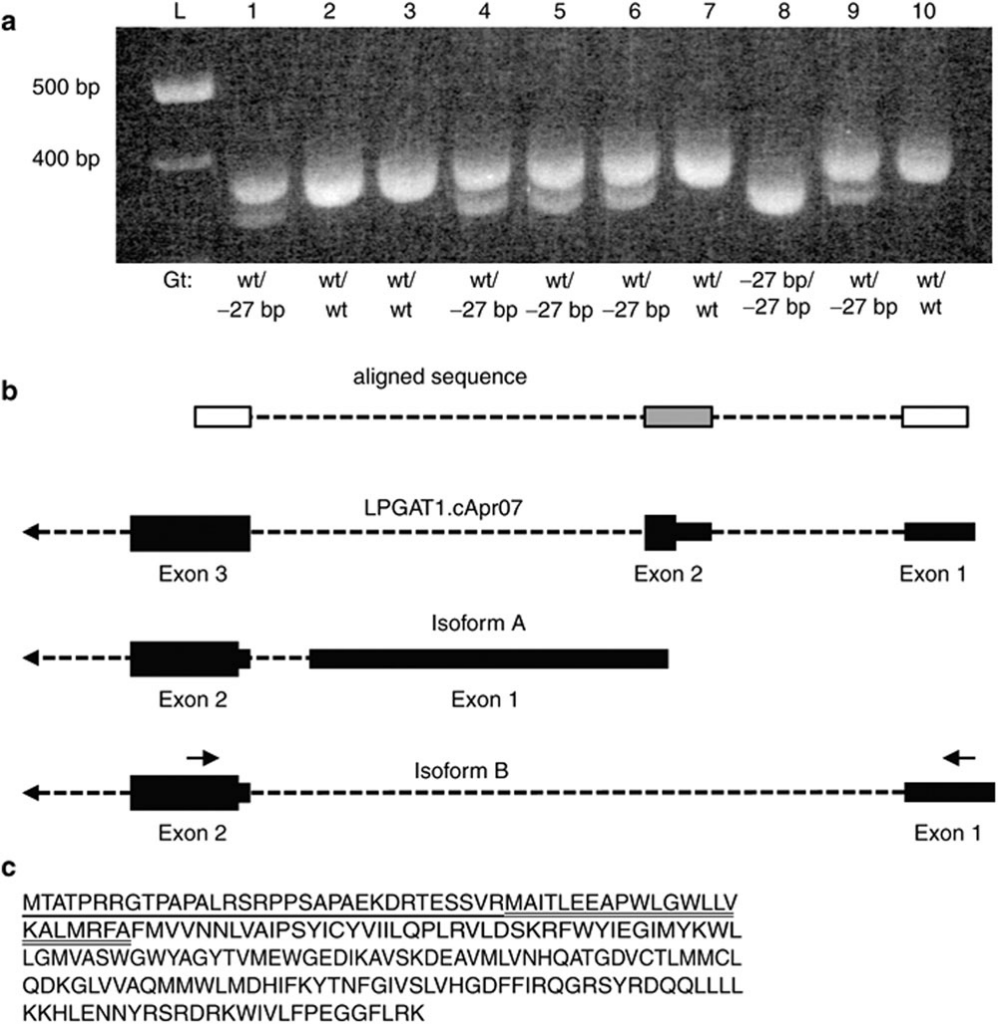
Identification of an alternative exon 2 in LPGAT1. (**a**) Gel image showing the results for the PCR reactions. PCR reactions were done using subcutaneous preadipocyte cDNA isolated from 10 Pima Indian subjects. (**b**) Schematic showing the alignment of the sequenced cDNA PCR products (aligned sequence) with the first three exons of the LPGAT1.cApr07 partial transcript (AceView Gene Models track, UCSC Genome Browser). The alternative exon identified by sequencing, which is also present in the LPGAT1.cApr07 transcript is highlighted by the gray shaded rectangle. For the LPGAT1.cApr07, isoform A, and isoform B transcripts the thicker bars indicate regions that are translated and the thinner bars indicate regions that are not translated. Horizontal arrows in exons 1 and 2 of isoform B show the locations of the primers used to screen the preadipocyte cDNA. (**c**) Amino acid sequences for the translated exons shown in (**b**). Single underline indicates the putative mitochondrial targeting peptide (exon 2 of LPGAT1.cApr07). The signal peptide was predicted using TargetP 1.1(25) and iPSORT(26) subcellular localization prediction programs. Double underline indicates the endoplasmic reticulum signal peptide for both isoforms A(15) and B. Gt, genotype; L, DNA ladder; LPGAT1, lysophosphatidylglycerol acyltransferase 1; wt, wild-type (no deletion).

To verify the presence of the 27bp deletion (LPGAT1-4) in the 5′-UTR of the *LPGAT1* isoform B mRNA transcript, subcutaneous preadipocyte cDNA previously isolated from 10 Pima Indian subjects was PCR amplified using primers located in exons 1 and 2 of isoform B and the PCR products were analyzed by gel electrophoresis. As expected, individuals homozygous for the 27bp deletion had a single amplified band running slightly lower than the band observed for individuals homozygous for the nondeleted mRNA transcript, whereas heterozygous individuals had both bands (Figure 2a). However, all of the PCR products were 148 bases larger than anticipated. Sequencing and alignment of the PCR products to the human genome (Human BLAT search, UCSC Genome Browser, Human March 2006 assembly) revealed that this cDNA matches a partial *LPGAT1* transcript (LPGAT1.cApr07) shown in the AceView gene models track, UCSC Genome Browser which differs from the *LPGAT1* isoform B in that it has an additional exon (148 bps) located between exons 1 and 2 (Figure 2b). Part of this alternative exon may be translated (Figure 2b, thicker region and Figure 2c, single underlined region) and the subcellular localization prediction programs TargetP 1.1 (27) and iPSORT (28) both predicted that the alternative N-terminal sequence could potentially serve as a mitochondrial targeting peptide (data not shown). For comparison, the N-terminal sequences for isoforms A and B (Figure 2b, exon 2 for both isoforms and Figure 2c, double underlined region) were also queried and both programs predicted that this region may function as an endoplasmic reticulum signal peptide (data not shown).

### Functional studies for the rs112662024 and LPGAT1-4 mutations

Rs112662024 and LPGAT1-4 (27bp deletion) were selected for further functional analysis based on their locations within the gene. The rs112662024 (G insertion) variant is located in a highly conserved region in the 3′-UTR 159 bases from the stop codon and 79 bases from a predicted miRNA binding site (Supplementary Figure S1a,b online) whereas the 27bp deletion is located in the 5′-UTR (exon 1) of isoform B in a region with strong regulatory potential (Supplementary Figure S1b online). To examine whether rs112662024 could possibly affect posttranscriptional regulation (e.g., mRNA stability, translation efficiency), genomic fragments with and without the G insertion (+G and −G, respectively; Supplementary Figure S1a online) were cloned into the pGL3-promoter vector at the 3′ end of the luciferase gene. Since 14 additional variants were also indentified in the 3′-UTR, only the first 402 bps were cloned in order to avoid those other variants. The two constructs (rs112662024^−G^ and rs112662024^+G^) and a no insert control (empty pGL3-promoter vector, pGL3-PV) were then co-transfected with pGL4.74[*hRluc*/TK] into HepG2, Caco-2, 3T3-L1, and G8 myoblast cell lines and luciferase activities were measured 48 h posttransfection. There was no significant difference in luciferase activity between the rs112662024^−G^ and rs112662024^+G^ constructs in any of the four cell lines tested (Supplementary Figure S1b-e online). However, in all four cell lines there was a significant decrease in luciferase activity for both constructs containing the rs112662024 inserts as compared with the pGL3-promoter vector control (no insert) (Supplementary Figure S1b-e online) suggesting that there may be a potential regulatory element located within this 402 bp region.

**FIGURE 3:**
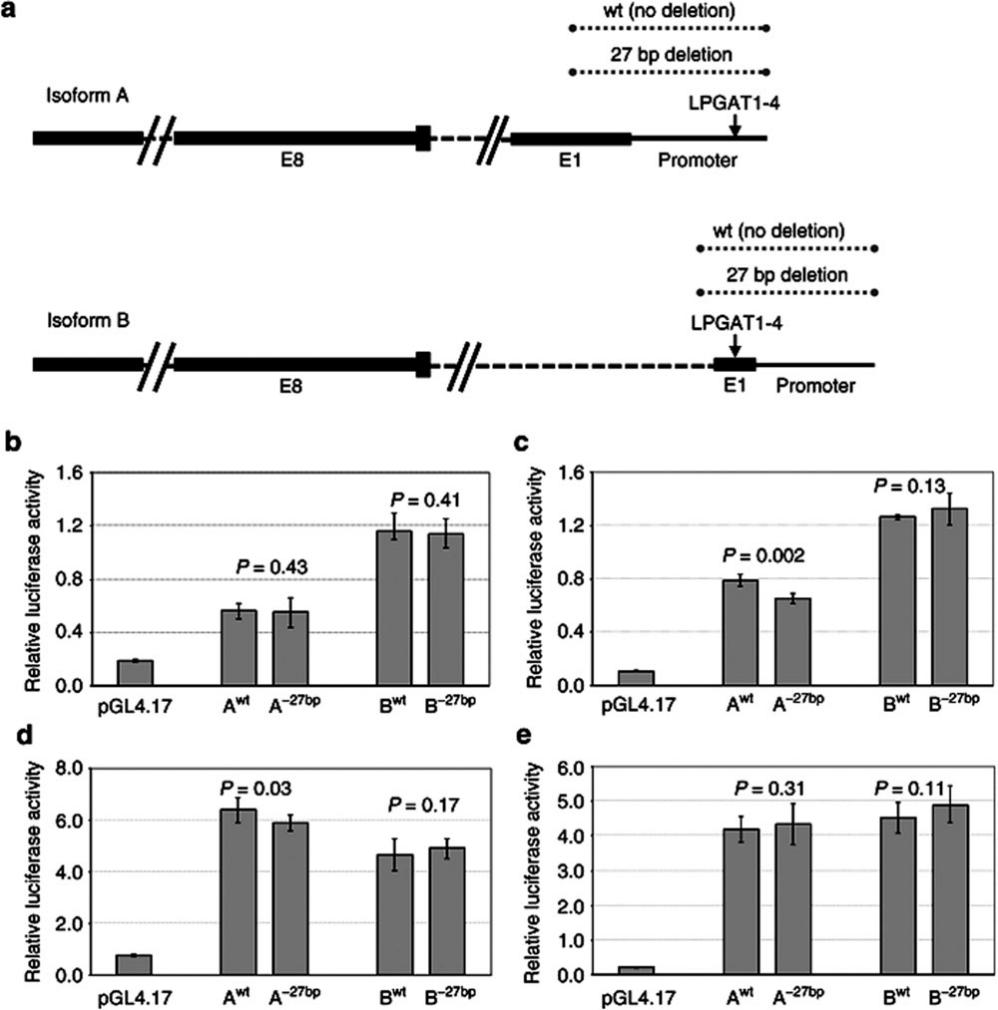
Functional studies to examine whether the 27 bp deletion affects transcriptional regulation. (**a**) Diagram showing the LPGAT1–4^wt^ and LPGAT1–4^−27 bp^ regions (dashed lines) that were cloned into the promoterless pGL4.17 vector. Relative luciferase activities for the LPGAT1–4^wt^ and LPGAT1–4^−27 bp^ isoform A and isoform B promoter constructs in (**b**) HepG2, (**c**) CaCo-2, (**d**) 3T3-L1, and (**e**) G8 myoblast cell lines. Transfections were done with equal amounts of plasmids. The constructs were co-transfected with pGL4.74[*hRluc*/TK] and luciferase levels are expressed as the relative activities and are shown as the means ± s.d. for six replicates. LPGAT1, lysophosphatidylglycerol acyltransferase 1; pGL3-BV, pGL3-basic vector no insert control; wt, wild-type (no deletion).

To determine whether the 27bp deletion (LPGAT1-4) affected a putative distal promoter element in isoform A or a promoter regulatory element in the 5′-UTR of isoform B, two sets of inserts without (wild-type) and with (−27bp) the deletion (Figure 3a) were cloned into the promoterless pGL4.17 vector. The constructs, isoform A^wt^, isoform A^−27bp^, isoform B^wt^, and isoform B^−27bp^, were co-transfected with pGL4.74[*hRluc*/TK] into HepG2, Caco-2, 3T3-L1, and G8 myoblast cell lines and luciferase activities were measured 48 h posttransfection. Both sets of LPGAT1-4^wt^ and LPGAT1-4^−27bp^ constructs had higher luciferase activities compared with the no insert control (pGL4.17) in all four cell lines (Figure 3b-e). However, there were only modest differences in luciferase activities between the isoform A^wt^ and isoform A^−27bp^ constructs in the Caco-2 and 3T3-L1 cells (Figure 3c,d), indicating that the 27bp deleted region is unlikely to significantly influence *LPGAT1* isoform A or isoform B promoter activity in these cell lines.

**FIGURE 4:**
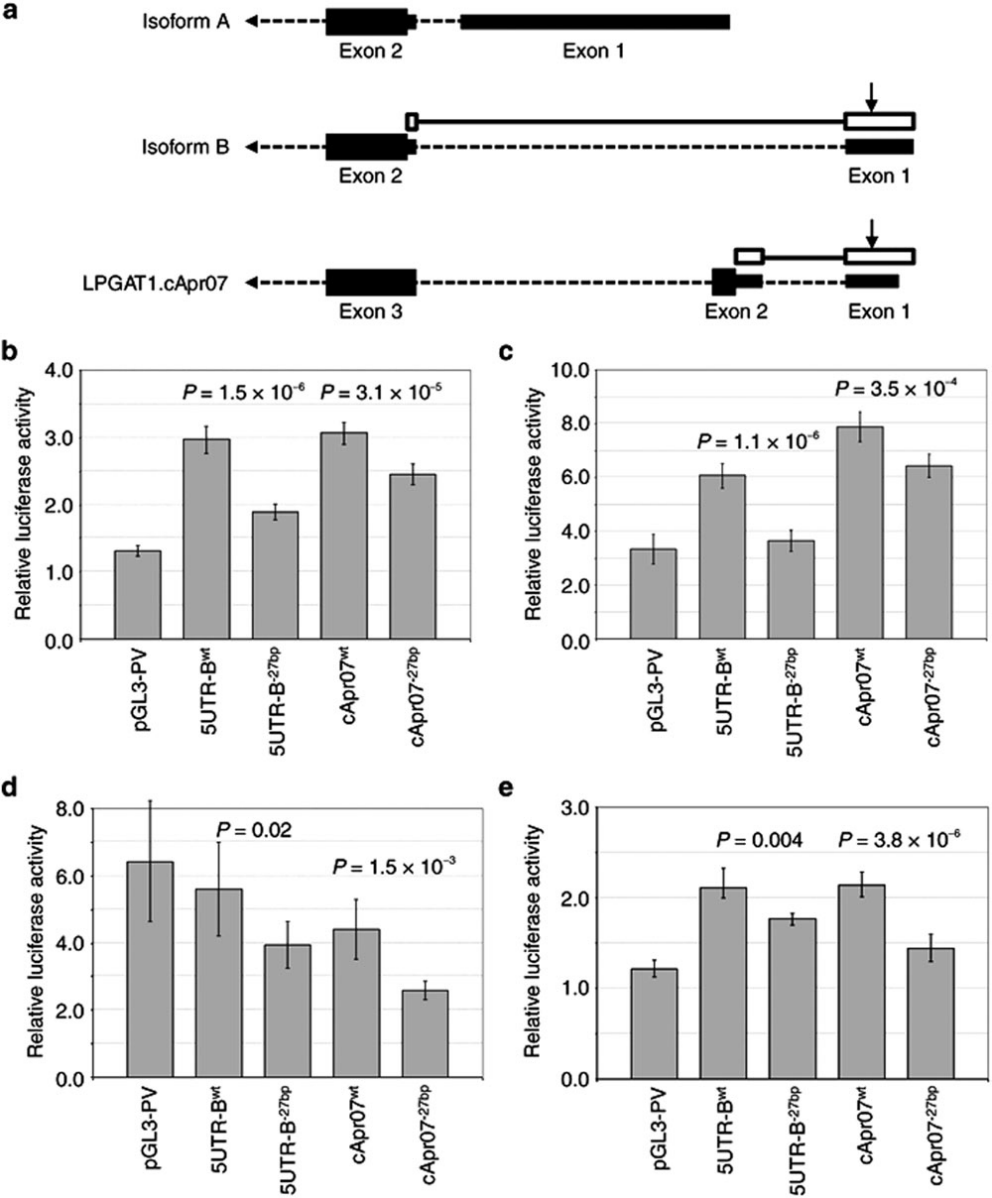
Functional studies to examine whether the 27 bp deletion affects posttranscriptional regulation. (**a**) Diagram showing the two *LPGAT1* 5′-UTRs (isoform B and LPGAT1.cApr 07) that were cloned into the pGL3-promoter vector. Open boxes indicate the regions that were cloned. Arrows indicate the location of the 27 bp deletion. Relative luciferase activities for the LPGAT1.5UTR-B^wt^, LPGAT1.5UTR-B^−27 bp^, LPGAT1.cApr07^wt^, and LPGAT1.cApr07^−27 bp^ constructs in (**b**) HepG2, (**c**) Caco-2, (**d**) 3T3-L1, and (**e**) G8 myoblast cell lines. Transfections were performed with equal amounts of plasmids. The constructs were co-transfected with pGL4.74[*hRluc*/TK] and luciferase assays were done in replicates of six and the results are shown as means ± s.d. LPGAT1, lysophosphatidylglycerol acyltransferase 1; pGL3-PV, pGL3-promoter vector no insert control; UTR, untranslated region; wt, wild-type (no deletion).

**FIGURE 5:**
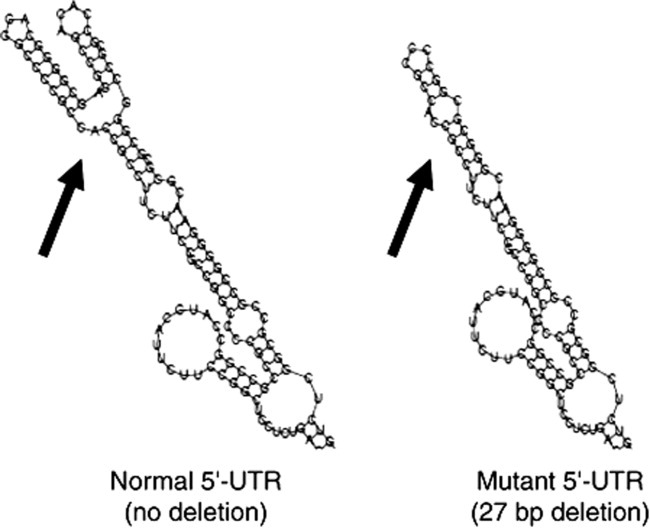
Predicted RNA secondary structures for both normal (no deletion) and mutant (27 bp deletion) transcripts. The 27 bp deletion eliminated a double-stem loop structure and a smaller single doublelooped region formed (arrows). Only the region that was altered is shown. The alteration was the same for both sets of transcripts (isoform B and LPGAT1.cApr 07). LPGAT1, lysophosphatidylglycerol acyltransferase 1; UTR, untranslated region.

Since the 27bp deletion is located in the 5′-UTRs of isoform B and LPGAT1.cApr07 (Figure 4a), the 5′-UTRs of both mRNAs without (wild-type) and with the deletion (−27bp) were cloned into the pGL3-promoter vector between the SV40 promoter and start codon of the luciferase gene to examine whether the deletion may potentially affect posttranscriptional regulation. The four constructs (LPGAT1.5UTR-B^wt^, LPGAT1.5UTR-B^−27bp^, LPGAT1.cApr07^wt^, and LPGAT1. cApr07^−27bp^) were co-transfected with pGL4.74[*hRluc*/TK] into HepG2, Caco-2, 3T3-L1, and G8 myoblast cell lines. For the HepG2, Caco-2, and G8 myoblast cell lines, the luciferase activities for the constructs containing the 5′-UTRs were higher compared with the empty vector control (pGL3-PV), and there was a moderate but statistically significant decrease in luciferase activities for the two 5′-UTR^−27bp^ constructs compared with the 5′-UTR^wt^ constructs (Figure 4b,c.e). For the 3T3-L1 cells, there was not an increase in luciferase activities for the 5′-UTR constructs compared with the pGL3-PV control, however, similar to the other three cell lines, there was still a modest but statistically significant decrease in luciferase levels for the 5′-UTR constructs containing the 27bp deletion (Figure 4c). These data suggest that the 27bp deletion may affect the mRNA for both LPGAT1 isoform B and LPGAT1.cApr07. The free energies and RNA secondary structures for the full length LPGAT1 isoform B and partial LPGAT1.cApr07 transcripts with and without the 27bp deletion were predicted using the RNAfold WebServer (29,30). For both sets of transcripts, there was a 17.5 unit change in the minimum free energy predictions (LPGAT1 isoform B^wt^, −1893.97 kcal/mol; LPGAT1 isoform B^−27bp^, −1876.47, and LPGAT1.cApr07^wt^, −401.72 kcal/mol; LPGAT1.cApr07^−27bp^, −384.22 kcal/mol); and, comparison of their predicted secondary structures show that the 27bp deletion results in the removal of two stem-loop structures attached to a larger loop, which is replaced by two small loops separated by one pair of nucleotides (Figure 5).

## Discussion

A GWAS for BMI in Pima Indians led us to analyze *LPGAT1* as a candidate gene for human obesity. Variants in this gene were nominally, but reproducibly associated with maximum BMI and repeated BMI measures in two nonoverlapping population-based samples from the Gila River Indian Community. As might be expected, these variants are also associated with percent body fat within a subset of individuals who have measures of body composition. We are not aware of any other published GWASs or large meta-analyses reporting an association between this locus and obesity. Our GWAS lead variant, rs4951552, whose major/minor alleles are opposite in Native Americans compared with Caucasians, also had a nominal association with both BMI and percent body fat (additive *P* = 0.03) in a GWAS of ∼1,000 Caucasian subjects (P. Kovacs, personal communication), but the obesity risk alleles were also flipped. However, the finding of a significant association between rs4951552 and BMI in Caucasians might not be expected because rs4951552 is unlikely to be a functional variant and the LD in this region is much higher in Pima Indians compared with Caucasians. In addition, our observation that two haplotypes, which only differ by the 27bp deletion (not present or very rare in Caucasians) are associated with either BMI in the full-heritage Pima Indians or mixed-heritage Native Americans suggests that either a rare functional variant is being captured by different haplotypes in different ethnic groups, or it suggests the presence of multiple functional alleles. We are in the process of obtaining whole genomic sequence data for Pima Indians to better evaluate the role of ethnic specific variants on BMI.

Haplotype 2, which showed the strongest association with BMI in the full-heritage Pima Indians, was the only haplotype associated with *LPGAT1* gene expression levels in muscle. Although a similar trend was observed between haplotype 2 and *LPGAT1* expression in adipose tissue, the association did not reach significance. The reason(s) for this difference is unclear. It could be an artifact (false positive or false negative) due to having a small number of tissue samples from individuals homozygous for haplotype 2 (muscle, *n* = 10; adipose *n* = 6, Figure 1). Alternatively, the tissue specific nature of the correlation may be driven by valid differences in biology. For example, *LPGAT1* isoform A has an alternative 5′-UTR that is located downstream of the region containing the 27bp deletion (Supplementary Figure S1 online and Figure 4), therefore differences in expression between the isoforms in different tissues may obscure an association with haplotype 2 and *LPGAT1* expression levels in adipose.

The 27bp deletion identified in the 5′-UTR (exon 1) of isoform B removes a predicted E2F (transcription factor important for adipogenesis) binding site, but no significant difference in transcriptional regulation was observed between the LPGAT1-4^wt^ and LPGAT1-4^−27bp^ isoform A and isoform B promoter constructs in the four cell lines examined in this study. However when the 5′-UTRs of *LPGAT1* isoform B and LPGAT1.cApr07 containing the 27bp deletion were cloned as the 5′-UTR for the luciferase gene, the deletion resulted in a decrease in luciferase activity compared with the no deletion controls suggesting that the deletion may affect posttranscriptional regulation. The deletion was also predicted to affect the free energies and secondary structures of the *LPGAT1* transcripts, and it may be these changes that are responsible for the decrease in luciferase levels either by affecting translation efficiency or mRNA stability. Further *in vitro* transcription/translation and mRNA decay experiments need to be done to address those possibilities.

A recent report demonstrated that LPGAT1 has monoacylglycerol acyltransferase like enzyme activity (16) and MGATs along with DGATs are involved in various lipid and energy metabolism related functions (4,16). It was observed that *LPGAT1* knockdown in the liver of *db*/*db* mice resulted in a decrease of serum TAG, total cholesterol, free fatty acids, and body weight, and therefore suggested that *LPGAT1* may be involved in liver TAG synthesis and secretion (16). A second group has also shown that LPGAT1 is localized in the endoplasmic reticulum and may be involved in the reacylation (remodeling) of LPG back to phosphatidylglycerol (15). Phosphatidylglycerol is a phospholipid with various biological roles including serving as a precursor for cardiolipin a major phospholipid found in the inner mitochondrial membrane and important for the proper function of enzymes involved in mitochondrial energy metabolism (15,31,32). It is also thought that remodeling of cardiolipin may generate pathological forms of the phospholipid, which may be responsible for the mitochondrial dysfunction seen with various diseases such as obesity and diabetes (32,33). In preadipocyte cDNA we observed an alternative exon located between exons 1 and 2 of isoform B that matched a partial *LPGAT1* transcript shown in the AceView gene models track (UCSC Genome Browser), which may contain a putative mitochondrial signaling peptide. Yang et al. (15), identified and characterized an alternative human *LPGAT1* gene (NCBI accession BC034621; isoform A, Supplementary Figure S1 online and Figure 5a) that contains a larger alternative exon 1 and they show that this LPGAT1 isoform is localized to the endoplasmic reticulum in Cos-7 cells (15). We also screened muscle cDNA isolated from several Pima Indian subjects (data not shown), and observed PCR products corresponding to both the partial AceView transcript containing the alternative exon, and to exons 1 and 2 of isoform B, which contains the same endoplasmic reticulum signaling peptide identified by Yang et al.(15). Based on these observations, it is possible that LPGAT1 may have multiple subcellular localizations (e.g., endoplasmic reticulum and mitochondrial) and therefore could potentially have multiple functions (e.g., LPGAT and/or monoacylglycerol acyltransferase activities) in different cells or within the same cells. Further fractionation and/or immunocytohistochemical studies need to be done to verify that LPAGT1 is indeed localized to the mitochondria in preadipocytes and other cell types.

In conclusion, we identified several variants in the *LPGAT1* region that are nominally but reproducibly associated with obesity in Pima Indians and other Native Americans. Functional studies suggest that the 27bp deletion in the 5′-UTR could possibly affect *LPGAT1* mRNA stability; however, our association studies suggest that the deletion is unlikely to be the only functional polymorphism in this region.
